# Computer-assisted lip diagnosis on traditional Chinese medicine using multi-class support vector machines

**DOI:** 10.1186/1472-6882-12-127

**Published:** 2012-08-16

**Authors:** FuFeng Li, Changbo Zhao, Zheng Xia, Yiqin Wang, Xiaobo Zhou, Guo-Zheng Li

**Affiliations:** 1 Laboratory of Information Access and Synthesis of TCM Four Diagnosis, Shanghai University of Traditional Chinese Medicine, Shanghai, 201203, China; 2 Department of Radiology, The Methodist Hospital Research Institute & Weill Cornell Medical College of Cornell University, Houston, TX, USA; 3 Department of Control Science & Engineering, Tongji University, Shanghai, 201804, China

**Keywords:** Traditional chinese medicine, Computer-assisted lip diagnosis, Image analysis, Feature selection, Support vector machine

## Abstract

**Background:**

In Traditional Chinese Medicine (TCM), the lip diagnosis is an important diagnostic method which has a long history and is applied widely. The lip color of a person is considered as a symptom to reflect the physical conditions of organs in the body. However, the traditional diagnostic approach is mainly based on observation by doctor’s nude eyes, which is non-quantitative and subjective. The non-quantitative approach largely depends on the doctor’s experience and influences accurate the diagnosis and treatment in TCM. Developing new quantification methods to identify the exact syndrome based on the lip diagnosis of TCM becomes urgent and important. In this paper, we design a computer-assisted classification model to provide an automatic and quantitative approach for the diagnosis of TCM based on the lip images.

**Methods:**

A computer-assisted classification method is designed and applied for syndrome diagnosis based on the lip images. Our purpose is to classify the lip images into four groups: deep-red, red, purple and pale. The proposed scheme consists of four steps including the lip image preprocessing, image feature extraction, feature selection and classification. The extracted 84 features contain the lip color space component, texture and moment features. Feature subset selection is performed by using SVM-RFE (Support Vector Machine with recursive feature elimination), mRMR (minimum Redundancy Maximum Relevance) and IG (information gain). Classification model is constructed based on the collected lip image features using multi-class SVM and Weighted multi-class SVM (WSVM). In addition, we compare SVM with k-nearest neighbor (kNN) algorithm, Multiple Asymmetric Partial Least Squares Classifier (MAPLSC) and Naïve Bayes for the diagnosis performance comparison. All displayed faces image have obtained consent from the participants.

**Results:**

A total of 257 lip images are collected for the modeling of lip diagnosis in TCM. The feature selection method SVM-RFE selects 9 important features which are composed of 5 color component features, 3 texture features and 1 moment feature. SVM, MAPLSC, Naïve Bayes, kNN showed better classification results based on the 9 selected features than the results obtained from all the 84 features. The total classification accuracy of the five methods is 84%, 81%, 79% and 81%, 77%, respectively. So SVM achieves the best classification accuracy. The classification accuracy of SVM is 81%, 71%, 89% and 86% on Deep-red, Pale Purple, Red and lip image models, respectively. While with the feature selection algorithm mRMR and IG, the total classification accuracy of WSVM achieves the best classification accuracy. Therefore, the results show that the system can achieve best classification accuracy combined with SVM classifiers and SVM-REF feature selection algorithm.

**Conclusions:**

A diagnostic system is proposed, which firstly segments the lip from the original facial image based on the Chan-Vese level set model and Otsu method, then extracts three kinds of features (color space features, Haralick co-occurrence features and Zernike moment features) on the lip image. Meanwhile, SVM-REF is adopted to select the optimal features. Finally, SVM is applied to classify the four classes. Besides, we also compare different feature selection algorithms and classifiers to verify our system. So the developed automatic and quantitative diagnosis system of TCM is effective to distinguish four lip image classes: Deep-red, Purple, Red and Pale. This study puts forward a new method and idea for the quantitative examination on lip diagnosis of TCM, as well as provides a template for objective diagnosis in TCM.

## Background

Lip diagnosis is one of the important methods in the clinical diagnosis of TCM. It is an approach to make disease diagnosis through observing the changes of lip complexion and understand the physiological functions and pathological changes of the body. The traditional lip diagnostic approach is mainly based on observing with nude eyes. The diagnostic results largely depend on personal clinical experience, and different TCM masters may deduce the different conclusion. Hence traditional lip diagnosis is not a quantitative or reliable approach which may be affected by many subjective factors. This study is to provide an automatic and quantitative approach to explore the diagnosis of Traditional Chinese Medicine (TCM) based on the lip images. Automated recognition based on the lip images, if proven accurate, can ultimately serve as an objective diagnostic way for TCM.

As a complete medical system, Traditional Chinese Medicine (TCM) plays an indispensable role in medical care in China. Different from the reductionism thinking mode of Western Medicine, TCM is based on the holistic and systematic ideas [1]. TCM practices are believed by many patients and scientists to be very effective, sometimes offering palliative efficiency when the practices of western medicine fail or are unable to provide treatment, especially for routine ailments such as flu and allergies, or Western medicine fails to relieve patients suffering from chronic ailments and functional disorders, such as migraines and osteoarthritis.

TCM diagnostics are mainly based on overall observation of human symptoms rather than “micro” level laboratory tests. There are four diagnostic methods in TCM: Inspection, Olfaction & auscultation, Interrogation and Palpation. According to inspection theory of TCM, the spleen opens in the mouth, the reflection in the lip. It is said that the lip color of a person reflects the healthy conditions of his organs etc. However, the traditional diagnostic approach is conducted by doctors mainly based on observing with nude eyes, description language, and experience discrimination. Due to lack of a standard evaluation criterion, the repeatability of the outcome is poor in the diagnosis of TCM. Therefore, with the help of modern computer science and information technology, developing new objective diagnosis method of traditional Chinese medicine which has comparable diagnosis accuracy with TCM veteran practitioner becomes urgent and important.

With the introduction of pattern recognition and data mining techniques, investigators have applied several algorithms to the research of diagnostic standardization and quantification in TCM. Such as Bayesian networks, Linear Discrimination Analysis, artificial neural networks, multi-label learning algorithm and image analysis technique etc. Chiu [2] built a computerized tongue examination system (CTES) based on computerized image analysis. The colors of the tongue and the thickness of its coating were identified using chromatic and textural algorithm. CTES was shown to be significantly consistent within itself with *P*<0.05 using the Hotelling multivariate statistical test. Zhang, et al. [3] used Bayesian networks to build tongue diagnosis model which provided a systematic and objective diagnostic standard for the tongue diagnosis. Li, et al. [4] used pattern recognition techniques for tongue diagnosis based on Linear Discriminated Analysis (LDA) on tongue diagnosis and achieved high accuracy in the quantitative diagnosis. Liu, et al. [5] introduced multi-label learning (MLL) into modeling of inquiry diagnosis for coronary heart disease (CHD) in traditional Chinese medicine. They demonstrated that the MLL techniques facilitated building standardized inquiry models in CHD diagnosis and showed a practical approach to solving the problem of labeling multi-syndromes simultaneously. However, this approach is designed based on the improved kNN approach. It only works in certain cases. In our study, this approach is slightly worse than other classifiers we applied. We firstly did the research on the computer-assisted diagnosis for the face inspection of TCM in 2006, we explored the collected and calculated method of face images, and initially developed TCM face images collecting system and set up 3600 cases database of face image from clinical patients [6-9] since then. Skin color has proven to be a useful and robust cue for face detection, localization and tracking, and a large number of skin color selection and classification have been reported in the last decade. Phung [10] studied various states-of-the-art skin modeling and classification algorithms. They reviewed methods in two surveys involving classifying individual pixels into skin and non-skin categories with a static model. All of those studies focus on two-class classification problem. Different from skin color classification, classifying lip images is a multi-classes problem according to TCM theory. Our research group [8,9] present some preliminary lip classify results based on SVM, in which some features of lips color in HSI color space were extracted to classify lip colors.

However, there is no systematic investigation about the different image features such as color space, texture and moment in the lip diagnosis of TCM. Meanwhile, the previous studies did not try to optimize the feature selection which is critical for our studies. In this study, we explore diagnosis of Traditional Chinese Medicine based on lip Images by combining imaging features from color component, textural and moment features, and assess the significance of each feature in classification. We apply the feature selection methods for the multi-class lip image classification. The proposed scheme consists of four major steps: image processing, feature extraction, feature selection and classification based on different algorithms. 10-fold cross validation is used to test the accuracy of the proposed classification scheme.

**Figure 1:**
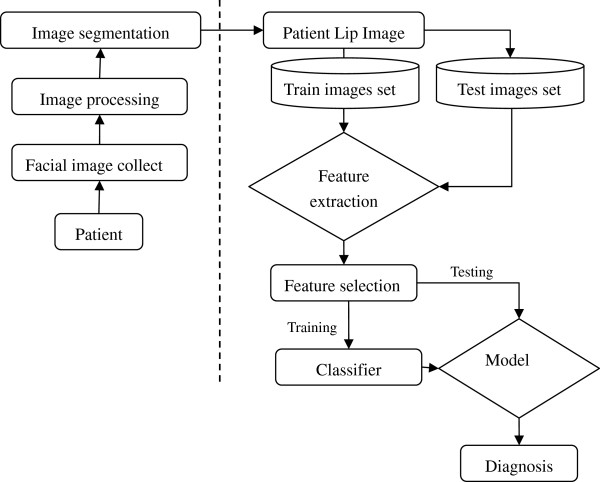
A framework of the proposed lip image classification model.

The developed system and the pipeline for automatically lip images identification are summarized in the Figure 1. The key components of the proposed system framework are facial image collection, image processing, image segmentation, lip image feature extraction, feature selection and classifier design. Firstly, facial image of patient is acquired through the facial image collection box composed of digital camera and LED light. After pre-processing and denoising, the facial image is segmented into forehead, left and right cheek, nose and lip. Finally we got the lip image before the image analysis. Then, extracting the color, texture and moment features for quantitative analysis the lip images. Next, to remove the irrelevant features and improve the performance of the learning system, we choose the sub-optimal features. Finally, a diagnosis modeling was built to distinguish different lip images in TCM. Our experimental results show the proposed system is an efficient tool for the lip images analysis using SVM.

The remaining of the paper is organized as follows: Section 2 describes our feature extraction, feature selection and classification methods. In Section 3, we present experimental results of the lip image classification model. Conclusions and directions for future research are presented in Section 4.

## Methods

Here we describe the developed system step by step. Firstly, we briefly describe the data sources and the image preprocessing techniques, and then we propose a SVM framework for the lip image classification in TCM by integrating color, texture and moment features into a pattern classification method. Before training the model, lip data is firstly normalized to have zero mean and unit variance. A feature selection method is then used to select a small set of effective features for classification in order to improve the generalization ability and the performance of the classifier. The data and the methodology are described next with more details.

### Data description and preprocessing

**Figure 2:**
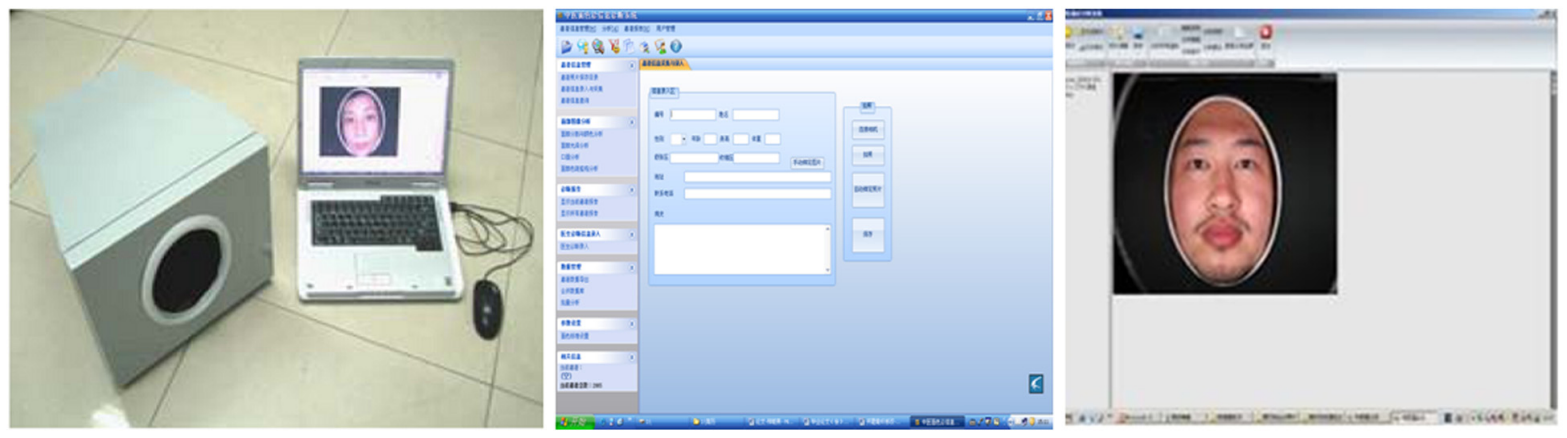
The face image (facial inspection) acquisition system.

We established the compatible face image acquisition system. It includes LED (the light source, color temperature value about 5600K, Ra = 90) and camera (the photographing medium). Its size is 36 cm × 40 cm × 28 cm. The other photographing conditions include distance 33 cm (between the camera and patients’ face), Tv (1/15 s), Av (5.6), ISO (80), white balance, custom mode and horizontal photography. The size of photographing windows was 220 mm x 170mm. As shown in Figure 2. The patients are selected in Longhua Hospital Affiliated to Shanghai University of Traditional Chinese Medicine, Shuguang Hospital Affiliated to Shanghai University of Traditional Chinese Medicine, Shanghai Renji Hospital. This study has been approved by the Shanghai society of medical ethics. All the patients have signed the informed consent form. The cases with incomplete information are removed. Three senior chief TCM physicians performed lip diagnosis individually for the patients, and the data with consistent results between 2 physicians are recorded. The physicians performed lip diagnosis using the clinical classification scale facial diagnosis of TCM (see Additional file 1). Finally, a total of 257 cases are obtained in the study. Among the 257 patients, 132 patients are female (51.4%), 125 patients are male (48.6%), with mean age of (55.39 ± 14.28). In this article, we extract the color component, texture and moment features from 257 lip images. Our data includes 90 images of Deep-red, 12 images of Pale, 62 images of Purple and 93 images of Red.

### Lip image segmentation

**Figure 3:**
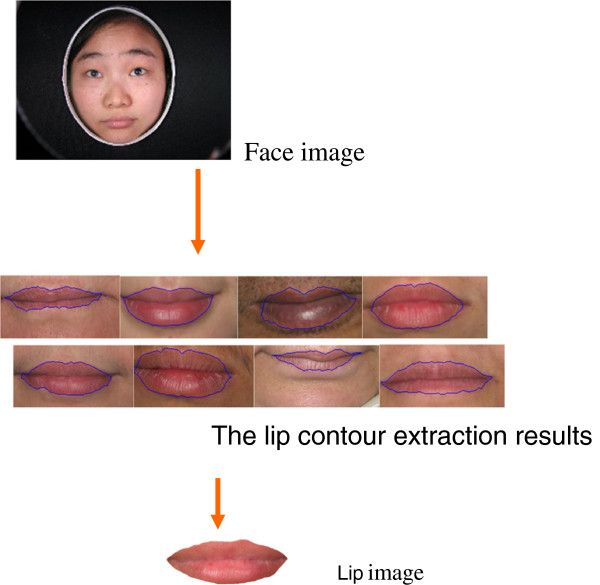
An example of lip image segmentation.

Accurate lip region segmentation is still a challenging and difficult problem due to the weak color contrast between lip region and non-lip region. To solve the problem, we developed a hybrid approach to automatically segment lip region with high precision based on Chan-Vese level set model and Otsu method, aimed to improve accuracy of automatic classification of lip diagnosis in TCM [10]. Firstly, mean-shift filter is used to smooth lip images. The difference of mean-shift filter and other filter methods is that mean-shift only smooth the same color region and not smooth the color change region. Secondly, a color space transformation that is fit for our lip segmentation is selected from some existing approaches. Lastly, level set image segmentation approach is used to get accurate lip region. An example of lip image segmentation is shown in the Figure 3. Experimental results on five hundred lip images show that the hybrid approach produces more accurate segmentation [11].

### Extraction of images features

We chose a large number of features (84) for investigation which included color, moments and texture features (see Additional file 2), as explained next.

#### *Color features*

The color space is a mathematical method which represents color. Colors have different quantitative methods. In HSI color space, a color is represented by a three dimensional vector corresponding to a position. I component represents intensity. H component represents hue. S component represents saturation. In RGB color space, a color can be obtained by mixing three basic colors-Red, Green, Blue different proportions. R, G, B is the range of 0 ~ 255. In the YCbCr color space, Y represents luminance, Cb and Cr represents color information. In YIQ color space, Y represents gray information, I and Q represents color information.

**Figure 4:**
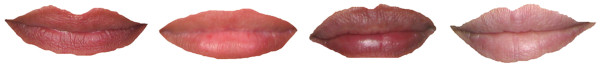
The lip color classification with nude eye.

The lip color is observed by TCM practitioner with nude eyes. Moreover, the lip color is mainly classified into Deep-red, Red, Purple and Pale etc. as shown Figure 4.

From intuition, the lip colors may be classifiable in RGB, HIS, YCbCr and YIQ color space. For instance, the colors “red” and “pale” are separable in the degree of intensity, and the colors “purple” and “deep red” are separable in the degree of Hue. So we select different color space as research content to extract feature.

In spite of the fact that the color histogram technique is a very simple and low-level method, it has shown good performance in practice especially for image indexing, retrieval and classification tasks, where feature extraction has to be as fast as possible.

The histogram features that we will consider are statistics based features. The extracting scheme is described as follows: 1) Lip images have been segmented from face images. It can determine the export lip area by removing the background. We can easily distinguish between the background and the lips because their colors are very different. When the background is white, which has the saturation value of 0.0, we can judge the value. In our experiment, a pixel is determined to be in the exit lip region if its saturation value is larger than 0.1. 2) To covert RGB representation of image to HIS, YcbCr, YIQ representation using different methods in image processing [12], we transform the Hue value from 0 to 360 degrees into interval from 0 to 1, to make the values in its components of HSI color space falling into range from 0 to 1. 3) The mean and variance of R, G, B, H, I, S, Y, Cb, Cr, Y, I, Q components are calculated in color space. After the above steps, we finally get 24 color component quantification values from the lip image.

#### *Haralick co-occurrence features*

As a traditional image feature extraction technique, the Haralick co-occurrence features use co-occurrence distribution of the gray image to generate the texture signature. Roughly speaking, given an offset on the image, the co-occurrence distribution of the image is referred to as the distribution of co-occurring values of pixel intensities. In our method, the Haralick co-occurrence features were extracted from each of the gray level spatial-dependence matrices [13]. The extracted 13 co-occurrence features were as follows: angular second moment, contrast, correlation, sum of squares, inverse difference moment, sum average, sum variance, sum entropy, entropy, difference variance, difference entropy, information measures of correlation, and maximal correlation coefficient [14].

#### *Zernike moment features*

Simply speaking, the Zernike moments features of an image are calculated based on the particular weighted averages of the intensity values. They are generated with the basic functions of Zernike polynomials. As classical image features, Zernike moments have wide applications [15]. Here, we give a brief description for calculating Zernike moments features for each lip image. First, calculate the center of mass for each lip image and redefine the lip pixels based on this center. Second, compute the radius for each lip, and define the average of the radii as *R*. Third, map the pixel $\left(x,y\right)$ of the lip image to a unit circle and obtain the projected pixel as $\left(x^{\prime },y^{\prime }\right)$. Since the Zerike moments polynomials are defined over a circle of radius 1, only the pixels $\left(x^{\prime },y^{\prime }\right)$ within the unit circle will be used to calculate Zerike moments. Let $I\left(x^{\prime },y^{\prime }\right)$ denotes the florescence of the pixel. The Zerike moment for each lip image is defined as:

$$\;Z_{\mathrm{nl}}=\frac{n+1}{\pi }\sum_{x}\sum_{y}V_{\mathrm{nl}}^{*}\left(x^{\prime },y^{\prime }\right)\cdot I\left(x^{\prime },y^{\prime }\right)$$

Where ${x^{\prime }}^{2}+{y^{\prime }}^{2}\leq 1$, 0≤l≤n, n−l is even, and $V_{\mathrm{nl}}^{*}\left(x^{\prime },y^{\prime }\right)$can be calculated as:

$$\;\begin{array}{cc} V_{\mathrm{nl}}^{*}\left(x^{\prime },y^{\prime }\right)= & \sum_{m=0}^{\left(n-1\right)/2}{\left(-1\right)}^{m}\frac{\left(n-m\right)!}{m!\left[\frac{n-2m+l}{2}\right]\left[\frac{n-2m-l}{2}\right]}\\ & x\;{\left({x^{\prime }}^{2}+{y^{\prime }}^{2}\right)}^{\left(n-2m\right)/2}e^{\mathrm{il\theta}} \end{array}$$

Finally, 47 moments features are obtained from the lip image.

### Feature selection

#### SVM-RFE

First the number of features is reduced by eliminating the less relevant features using a forward selection method based on a ranking criterion and then backward feature elimination is applied using a feature subset selection method, as explained next.

##### Features subset selection method based on SVM

The support vector machine recursive feature elimination (SVM-RFE) algorithm [16] is applied to find a subset of features that optimizes the performance of the classifier. This algorithm determines the ranking of the features based on a backward sequential selection method that removes one feature at a time. At each time, the removed feature makes the variation of SVM-based leave-one-out error bound smallest, compared to removing other features.

To remove the irrelevant features and improve the performance of the learning system, a prediction risk-based feature selection method is employed to choose the sub-optimal feature sets [16]. This method employs an embedded feature selection criterion of prediction risk, which evaluates features by calculating the change if the corresponding feature is replaced by its average value. It has several advantages. (1) The embedded feature selection model depends on learning machines. It can reach higher accuracy than the filter model, as well as keep the lower computation complexity than the wrapper model. (2) The prediction risk criterion had been employed with several different learning machines and outperformed Optimal Brain Damage when using multi-class SVM to test on more than 10 University of California Irvine (UCI) datasets [17].

#### mRMR

Most used filter type methods is to simply select top-ranked features without considering relationships among features. The minimum Redundancy Maximum Relevance (mRMR) algorithm [18] is also applied not only to realize max-dependency, but also to consider eliminating the redundancy features. The optimization of scoring functions are all based on mutual-information.

#### IG

Information gain (IG) is an alternative synonym for Kullback–Leibler divergence, which is frequently employed in the field of machine learning, especially in text categorization [19]. It measures the number of bits of information obtained for category prediction by knowing the presence or absence of a term in a document. Through IG algorithm for feature selection, the data must be discretized first before the experiment.

Classification is performed by following 50 times 10-fold cross validation on the training samples. Feature ranking based leave-one-out is performed using data. The feature selection method is implemented in each training subset in order to correct for the selection bias [20]. It is important that cross-validation is external to the feature selection process in order to more accurately estimate the prediction error. We combine the rankings of all leave-one-out experiments and report the total rank of features (in Figure 6 and Additional file 3).

**Figure 5:**
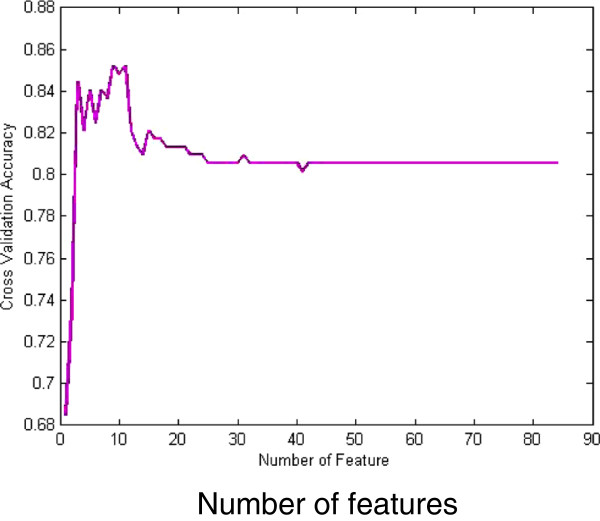
Cluster gram of the lip data.

### Classification

In recent years, many novel algorithms are proposed or improved based on typical classifiers. Just as the literature [21] proposed a quick SVM-based multi-class classifier, and proved that is effective and fast than some typical approaches such as one-versus-all and one-versus-one. To improve the performance of kNN, a k-NS classifier [22] is designed based on the distance from a query sample to the nearest subspaces, which is spanned by k nearest samples of the same class. Although the state-of-art classifiers are attractive, we still choose the typical SVM one-versus-one classifier and kNN due to the following reasons: (1) The lip image dataset we designed is a small-scale set, so the algorithm time-consuming problem is not our main consideration. (2) Most TCM frameworks adopt these typical classifiers for classification, yet with extracting different types of features [8]. Thus the algorithms in our framework need to be compared on similar classifiers and different features. That will support our opinions and arguments with the extracted three features which are more effective in this paper. However, it is extremely significant to consider these state-of-art methods in our future work which may evolve into a large-scale problem and bring in a novel framework concerned more about the real-time problem.

#### SVM

Classification is performed by starting with the more discriminative features and gradually adding less discriminative features, until classification performance no longer improves. Support Vector Machines [23,24] with poly kernel is used as classifier. We map the input vectors into a feature space, either linearly or non-linearly, which is relevant to the selection of the kernel function. Then, within the feature space, seek an optimized linear division; i.e. construct a hyper-plane that can separate the entire samples into two classes with the least errors and maximal margin. The SVM training process always seeks a global optimized solution and avoids over-fitting, so it has the ability to deal with a large number of features.

#### Weighted-SVM (WSVM)

Since the data are highly unbalanced and the sample size is rather small to produce balanced classes by sub sampling the largest class, we used a weighted SVM [25-27] to apply larger penalty to the class with the smaller number of samples. If the penalty parameter is not weighted (equal *C* for both classes), there is an undesirable bias towards the class with the large training size; and thus we set the ratio of penalties for different classes to the inverse ratio of the training class sizes.

#### Multi-class SVM

The multi-class problem is solved by constructing and combining several binary SVM classifiers into a voting scheme. We apply majority voting from one-versus-one classification problems. The predictive ability of the classification scheme is assessed by 10-fold cross validation.

#### MAPLSC

The Multiple Asymmetric Partial Least Squares Classifier (MAPLSC) [28] is an extension of classifier APLSC to the multi-class problem, and APLSC is an asymmetric PLS classifier, which sophisticatedly researches into the imbalanced distribution between classes. MAPLSC adopted the pairwise coupling strategy for combining the probabilistic outputs of all the one-versus-one binary classifiers to obtain estimates of the posterior probabilities for all candidate classes.

As a performance comparison, we also consider the Naïve Bayes classifier and the kNN classifier.

A classifier can provide a criterion to evaluate the discrimination power of the features for the feature subset selection. A kNN classifier is chosen for its simplicity and flexibility. Each sample is represented as a vector in an n-dimension feature space. The distance between two samples is defined by the Euclidian distance. A training set is used to determine the class of a previously unseen nucleus. The classifier calculated the distances between one sample and the others in the training set. Next, the K samples in the training set which are the closest to the unseen samples are selected. The class label of this sample is determined to be the class of the most common sample type in the K nearest neighbors. For Naïve Bayes classifier, it is a simple probabilistic classifier based on the Bayes rule and assumes that feature variables are unrelated to any other features. Despite the conditional independence assumptions, Naïve Bayes classifiers also have good classification performance for many complex real-world datasets.

All the above classifiers are realized in MATLAB toolboxes, SVM-KM [23] and LIBSVM [27]. Simultaneously, they can also be carried out with other libraries, such as LIBLINER [29], which is alternative for SVM classification, especially appropriate for large-scale classification problems.

## Results

Prior to classification of lip image, we use segmentation algorithm based on level set model to get the lip images in face. In order to evaluate the quantification effect of the experiments, the clinical experts have classified the lip images based on color, and the results of the lip images clinical experts determined are consistent. In this study, lip images are divided into 4 categories: Deep-red, Red, Purple and Pale.

### Clustering analysis of data

The quantified features are first investigated by using hierarchical clustering approach. This clustering procedure produces a heat map with dendrogram by Hierarchical clustering. The clustering on the all samples is shown in Figure 5 where the horizontal coordinate stands for the clustering of the samples and the vertical coordinate represents the features. We can clearly see some distinguished patterns among the samples and features. This result demonstrates that the lip images are separable.

**Figure 6:**
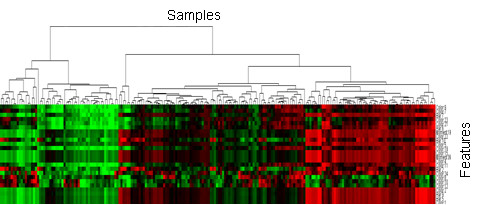
The trend of the cross validation accuracy versus retained feature number by SVM-RFE.

### Results of feature selection

The results of feature selection using SVM-RFE are shown in Figure 6. The aim of feature selection is to reduce dimension of the lip image diagnosis of TCM and to find the most related symptom subsets. In order to incorporate WSVM, MAPLSC, Naïve Bayes and kNN into SVM-RFE, lip datasets need to select the sub-optimal features before the use of classifiers. SVM-RFE first removes the smallest sorting coefficient feature which is constructed by the weight vector according to the SVM training result, then the rest features will be retrained through SVM to obtain the new sorting coefficient. After this iterative process, a feature ranking table is applied to define several feature subsets for SVM training. Finally, the optimal feature subset is selected on the basis of forecast accuracy. The result is seen that the performance is good when then the number of features is between 3 and 11. The most important 9 features are obtained after feature selection. They are comprised of 5 color component features, 3 texture features and 1 moment feature. According to the 9 selected features by SVM-RFE, each classifier will employ it to the training data in the classification algorithm before. Furthermore, our classification results demonstrate that the forecast accuracy is improved on the optimized 9-feature subset either by SVM, MAPLSC, Naïve Bayes and kNN. The selected features effect on the accuracy due to eliminate redundancy among features, in other words, it strengthens the correlation between features and its labels. Intuitively, it performs better on the classifier with the optimal subsets. Ranked feature list is shown in Additional file 3.

**Figure 7:**
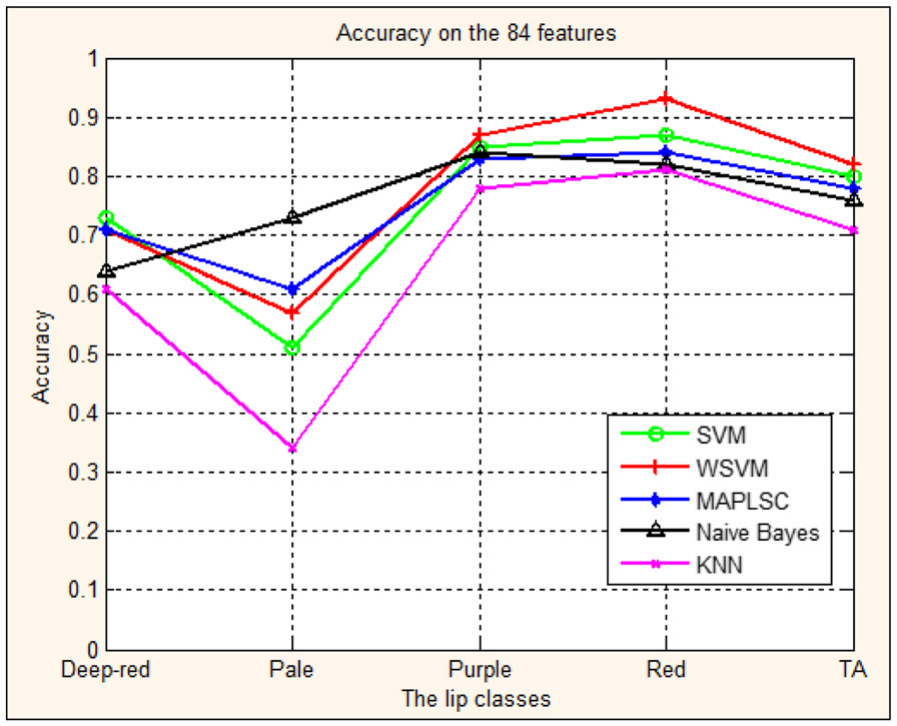
Prediction accuracy of lip image classification using SVM, WSVM, MAPLSC, Naïve Bayes and kNN on all the 84 features.

In addition, we compared SVM-RFE with filter type methods, mRMR and IG. Filter methods consider the feature selection problem as a ranking problem independently of the classifier. Furthermore, with cross validation in our experiment, mRMR and IG are used for each training data and can search the feature ranking according to their own search strategy. Meanwhile, Combined with SVM for evaluating the optimal feature subsets, we select approximately an average of 20 features on the mRMR and 26 features on the IG. Their results show that the WSVM can perform best on the total accuracy.

The feature selection and ranking showed that parameters extracted from lip image performed well for most classification tasks. Intuitively, the color features are closely associated with the lip classification. Our results demonstrate that moment and texture features also seemed to play important roles in lip classification.

#### Prediction results of the lip image classification on all the 84 features

Figure 7 and Table 1 show the classification accuracy of four lip image models with 84 features in inspection diagnosis of TCM. We repeated 10-fold cross validation 50 times, and then calculate the average accuracy and the variance of accuracy.

**Figure 8:**
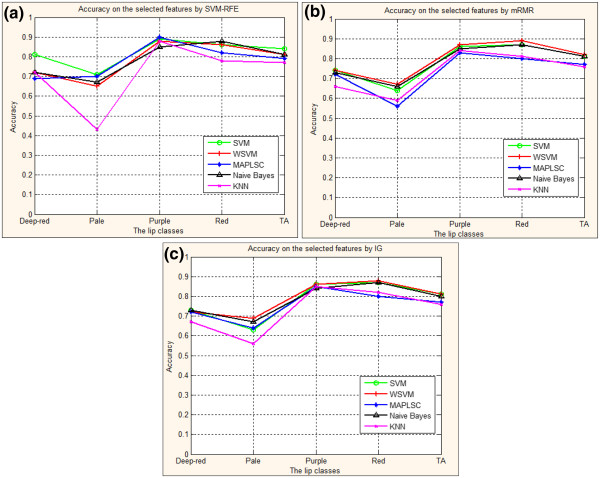
**(a). Prediction accuracy of lip image classification using SVM, WSVM, MAPLSC, Naïve Bayes and kNN on the 9 selected features by SVM-RFE. **(**b**): Prediction accuracy of lip image classification using SVM, WSVM, MAPLSC, Naïve Bayes and kNN on the selected features by mRMR. (**c**): Prediction accuracy of lip image classification using SVM, WSVM, MAPLSC, Naïve Bayes and kNN on the selected features by IG.

**Table 1:** Accuracy of lip image classification using SVM, WSVM, kNN,MAPLSC and Naive Bayes on all the 84 features (mean ± variance)

**Lip classes**	**SVM**	**WSVM**	**MAPLSC**	**Naïve Bayes**	**kNN**
Deep-red	0.73 ± 0.02	0.72 ± 0.02	0.71 ± 0.02	0.64 ± 0.02	0.61 ± 0.03
Pale	0.51 ± 0.22	0.57 ± 0.22	0.61 ± 0.22	0.73 ± 0.18	0.34 ± 0.20
Purple	0.85 ± 0.02	0.87 ± 0.02	0.83 ± 0.02	0.84 ± 0.02	0.78 ± 0.03
Red	0.87 ± 0.01	0.93 ± 0.01	0.84 ± 0.01	0.82 ± 0.02	0.81 ± 0.02
TA	0.80 ± 0.01	0.82 ± 0.01	0.78 ± 0.01	0.76 ± 0.01	0.71 ± 0.01

The accuracies on the 4 lip image classes of SVM, WSVM,MAPLSC, Naïve Bayes and kNN are also shown in Figure 5, where the horizontal axis stands for 4 classes of lip image forecasted and TA means the total accuracy of prediction results on the whole lip images; the vertical axis stands for prediction accuracy with 100% as the highest value.

The results in Figure 7 and Table 1 demonstrate that:1) The total accuracy of the whole lip images models using SVM, WSVM, MAPLSC, Naïve Bayes and kNN is 80%, 82%, 78% and 76%,71%, respectively. Comparing the total accuracy of the lip images models using WSVM with those using SVM, MAPLSC, Naïve Bayes, kNN, the total accuracy of WSVM is 11%, 6%, 4% and 2% higher than that of kNN, Naïve Bayes, MAPLSC, SVM. 2) On each class, SVM, WSVM, MAPLSC and Naïve Bayes obtain better results than kNN, three out of four lip classes of SVM, WSVM MAPLSC and Naïve Bayes have better prediction accuracies, i.e. the accuracy of SVM is 73%, 85%, 87% in Deep-red, Purple and Red model. 3) The forecast results vary on different classes. Although there are significant clinical values on the total accuracy on the whole samples, the best accuracy of Pale model is only 73% which needs to be further addressed in the future. From the above discussions, we find that, in many cases, the performance of SVM (or WSVM) achieves the best or the second best accuracy among all the classifiers.

#### Prediction results of the lip image classification on the selected features

**Figure 9:**
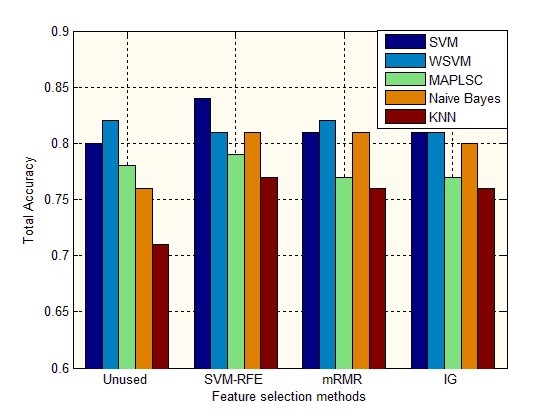
Perfection accuracy of lip image classification on different feature selection methods using SVM, WSVM, MAPLSC, Naïve Bayes and kNN.

Now we present the prediction result on the 9 selected features to illustrate the importance of feature selection in terms of accuracy improvement. Figure 9 and Table 2, Table 3, Table 4 show the forecast results for each lip class on the optimal subset features selected by SVM_RFE, mRMR, IG, respectively.

**Table 2:** Accuracy of lip image classification using SVM, WSVM, kNN,MAPLSC and Naive Bayes on the features selected by SVM-RFE (mean ± variance)

**Lip classes**	**SVM**	**WSVM**	**MAPLSC**	**Naïve Bayes**	**kNN**
Deep-red	0.81 ± 0.02	0.72 ± 0.02	0.69 ± 0.02	0.72 ± 0.03	0.72 ± 0.02
Pale	0.71 ± 0.16	0.65 ± 0.21	0.70 ± 0.22	0.67 ± 0.20	0.43 ± 0.22
Purple	0.89 ± 0.02	0.88 ± 0.02	0.90 ± 0.01	0.85 ± 0.02	0.88 ± 0.02
Red	0.86 ± 0.01	0.86 ± 0.01	0.82 ± 0.02	0.88 ± 0.02	0.78 ± 0.02
TA	0.84 ± 0.01	0.81 ± 0.01	0.79 ± 0.01	0.81 ± 0.05	0.77 ± 0.01

**Table 3:** Accuracy of lip image classification using SVM, WSVM, kNN, MAPLSC and Naïve Bayes on the features selected by mRMR (mean ± variance)

**Lip classes**	**SVM**	**WSVM**	**MAPLSC**	**Naïve Bayes**	**kNN**
Deep-red	0.74 ± 0.02	0.74 ± 0.02	0.72 ± 0.02	0.73 ± 0.02	0.66 ± 0.02
Pale	0.64 ± 0.21	0.67 ± 0.20	0.56 ± 0.23	0.66 ± 0.20	0.59 ± 0.20
Purple	0.86 ± 0.02	0.87 ± 0.02	0.83 ± 0.02	0.85 ± 0.02	0.84 ± 0.02
Red	0.87 ± 0.01	0.89 ± 0.01	0.80 ± 0.02	0.87 ± 0.01	0.81 ± 0.02
TA	0.81 ± 0.01	0.82 ± 0.01	0.77 ± 0.01	0.81 ± 0.01	0.76 ± 0.01

**Table 4:** Accuracy of lip image classification using SVM, WSVM, kNN, MAPLSC and Naïve Bayes on the features selected by IG (mean ± variance)

**Lip classes**	**SVM**	**WSVM**	**MAPLSC**	**Naïve Bayes**	**kNN**
Deep-red	0.73 ± 0.02	0.72 ± 0.02	0.72 ± 0.02	0.73 ± 0.02	0.67 ± 0.02
Pale	0.63 ± 0.21	0.69 ± 0.20	0.64 ± 0.23	0.67 ± 0.20	0.56 ± 0.20
Purple	0.86 ± 0.02	0.86 ± 0.02	0.85 ± 0.02	0.84 ± 0.02	0.85 ± 0.02
Red	0.87 ± 0.01	0.88 ± 0.01	0.80 ± 0.02	0.87 ± 0.01	0.82 ± 0.02
TA	0.81 ± 0.01	0.81 ± 0.01	0.77 ± 0.01	0.80 ± 0.01	0.76 ± 0.01

Results in Figure 9 and Table 2, Table 3, Table 4 show that: 1) The total accuracy of the whole lip images models on the subset of 9 features selected by SVM-RFE using SVM, WSVM, MAPLSC, Naïve Bayes and kNN is 84%, 81%, 79%, 81%,77% respectively. Comparing to the total accuracy on all the 84 features, the results of SVM, MAPLSC, Naïve Bayes, and kNN on the selected features are higher. And with mRMR and IG methods, the total accuracy has also improved on the SVM, Naïve Bayes and kNN. 2) Besides, the accuracies on each class by using mRMR and IG are more stable than that by using SVM-RFE, due to the generalization ability of filter method, as shown in Figure 8 (b)(c). Meanwhile, through the Table 3 and Table 4, we can observe that the accuracies are similar to each other, the reason is that mRMR and IG are all involved with information entropy theory. On the other hand, that is the particularity of the traditional Chinese medicine data. (3) The accuracy of SVM is 81%, 89%, 86% and 71% on Deep-red, Purple, Red and Pale lip image model respectively after using SVM-RFE. In other two methods mRMR and IG, the SVM also obtains higher accuracies than all the 84 features.4) Different feature subsets lead to different forecast accuracies. SVM is rather stable without much fluctuation than other classifiers. But whatever the subset is, SVM is superior to kNN and MAPLSC, especially on the subset of 9 features where SVM is better than MAPLSC, Naïve Bayes and kNN, and similar to WSVM.

Figure 9 demonstrates that different feature subsets lead to different forecast accuracy. The total accuracy on the subset of 9 features is 84% by SVM, 81% by WSVM, 79% by MAPLSC, 81% by Naïve Bayes and 77% by kNN. Comparing to the total accuracy on the subset of 84 features and the other two feature selection mRMR and IG, the results using SVM, MAPLSC, Naïve Bayes and kNN on the 9 selected features obtain higher accuracies than others.

After the detailed descriptions of classification results on the selected features, it shows that feature selection would take significant effect on the lip image data, especially the SVM-RFE adopted in our system. On the other hand, SVM and WSVM always obtain the top two accuracies comparing to others under different feature selection methods. SVM combined with SVM-REF would be the most efficient approach in our system.

Furthermore, in order to validate the statistical significance of the total accuracies on the five classifiers by using 50 times10-fold cross validation, the P value of statistical comparison as shown in Table 5. And a “greater than” symbol “>” is defined on the set of all comparing algorithms for each feature selection. A > B means that the performance of algorithm A is statistically better than that of algorithm B, which is based on 5% significance level. Note that it is possible that A > B in terms of some feature selection but B > A in terms of other ones. Therefore, we adopted pairwise comparison on all classifiers, and if A > B, then A got score +1 and B got scores −1 [30]. Based on the accumulated score of each algorithm on all feature selections, a total rank is defined to demonstrate the superiority of algorithms as shown in the last of Table 5. According to the Table 5, we have reason to believe that the results are statistically significant. And the classifiers WSVM and SVM perform better than others.

**Table 5:** P value of statistical comparisons among the five classifiers on the lip images data

** Feature selection methods**	** Classifier algorithm**
	** A-SVM; B-WSVM; C-MAPLSC; D-Naïve Bayes; E-kNN**
Unused	B > A(P = 3.2 × 10^-7^), A > C(P = 2.3 × 10^-6^), A > D(P = 1.8 × 10^-19^)
	A > E(P = 1.5 × 10^-61^), B > C(P = 8.5 × 10^-21^),B > D(P = 1.5 × 10^-40^)
	B > E(P = 2.7 × 10^-90^), C > D(P = 3.2 × 10^-6^), C > E(P = 2.2 × 10^-36^)
	D > E(P = 4.5 × 10^-17^)
SVM-RFE	A > B(P = 1.3 × 10^-16^), A > C(P = 3.5 × 10^-26^), A > D(P = 7.0 × 10^-14^)
	A > E(P = 5.1 × 10^-50^), B > C(P = 3.3 × 10^-3^), B > E(P = 2.6 × 10^-13^)
	D > C(P = 1.4 × 10^-4^), C > E(P = 6.2 × 10^-6^), D > E(P = 1.4 × 10^-16^),
mRMR	B > A(P = 5.1 × 10^-3^), A > C(P = 1.2 × 10^-18^), A > E(P = 6.0 × 10^-28^)
	B > C(P = 1.5 × 10^-28^), B > D(P = 3.1 × 10^-3^), B > E(P = 6.2 × 10^-40^)
	D > C(P = 5.7 × 10^-17^), C > E(P = 2.5 × 10^-2^),D > E(P = 1.5 × 10^-25^)
IG	A > C(P = 5.9 × 10^-11^), A > E(P = 6.2 × 10^-19^), B > C(P = 1.3 × 10^-15^)
	B > D(P = 1.9 × 10^-2^), B > E(P = 5.0 × 10^-25^), D > C(P = 3.1 × 10^-9^)
	C > E(P = 9.3 × 10^-3^), D > E(P = 1.7 × 10^-16^)
Total Rank	WSVM(11) > SVM(9) > Naïve Bayes(1) > MAPLSC(−6) > kNN(−16)

## Discussions

In the long time of clinical practice, a special diagnosis and therapy system has been formed according to the traditional TCM theory. Lip diagnosis is one of the special diagnostic methods in the clinical diagnosis of TCM. The lip information is mainly acquired by the doctors’ nude eyes. The diagnostic results largely depend on personal clinical experience, and different TCM masters may deduce the different conclusion. Hence traditional lip diagnosis is not a quantitative or reliable approach which may be affected by many subjective factors. This study is to introduce a quantitative and automatic approach to explore the diagnosis of TCM based on the lip images.

There are four steps in our new method including the lip image preprocessing, image feature extraction, feature selection and classification. The feature subset selection is performed by using SVM-RFE and IG. The classification model is constructed by using multi-class SVM and Weighted multi-class SVM (WSVM). All displayed lip images are satisfactory. A total of 257 lip images are collected for the modeling of lip diagnosis in TCM. The feature selection method SVM-RFE selects 9 important features which are composed of 5 color component features, 3 texture features and 1 moment feature. We may conclude that feature selection would take significant effect on the lip image date through our detailed descriptions of classification results. And SVM, MAPLSC, Naïve Bayes, kNN showed better classification results based on the 9 selected features than the results obtained from all the 84 features. The total classification accuracy of the five methods is 84%, 81%, 79% and 81%, 77%, respectively. So SVM achieves the best classification accuracy. The classification accuracy of SVM is 81%, 71%, 89% and 86% on Deep-red, Pale Purple, Red and lip image models, respectively. As a result, SVM turns out to be effective technique for solving problems with multi-class in clinical practice of TCM. Furthermore, combination of symptom selection with multi-class SVM algorithms decreases the dimension features in lip images diagnosis of TCM and consequently simplifies the feature information and increases forecast accuracy. Our results demonstrated that this method may be an objective diagnostic way for TCM in order to improve the clinical diagnosis of TCM.

## Conclusions

In this study, we developed a computer-assisted lip diagnosis system by combining color, Haralick co-occurrence and moment features in lip image. We exploited the potential of features extracted automatically from images and investigated the diagnostic value of each feature by applying the support vector machine recursive feature elimination algorithm. We also compare the above algorithm and scheme with many other feature selection methods and classification schemes. The developed automatic and quantitative diagnostic system of TCM is effective to distinguish four lip image classes: Deep-red, Purple, Red and Pale.

A multi-class SVM algorithm is employed to construct the lip inspection models for diagnosis of TCM, and further produces better results than kNN MAPLSC and Naïve Bayes on the optimal subset of 9 features. Meanwhile, with other filter type methods, it also shows that feature selection has been effective on the lip images datasets. Furthermore, combination of symptom selection with multi-class SVM algorithms decreases the dimension features in lip images diagnosis of TCM and consequently simplifies the feature information and increases forecast accuracy. The optimal feature subset obtained by feature selection could also be used for interpretation and guidance in clinical practice.

 Future works are to set up data collection more reasonably and effectively based on original image guided by the concept of face inspection, as well as to design more effective symptom selection algorithms, and to apply the multi-class SVM algorithms on more biomedical data sets. Besides, reference [31] is a related study of automatic diagnosis of TCM, which utilizes raw free-text clinical records for clinical practice. Compared to our well-structured lip image dataset through manually collected, this framework may provide an innovative idea to improve our diagnosis system.

## Competing interests

We, the authors, have no competing interests related to the manuscript entitled ”Computer-assisted Lip Diagnosis on Traditional Chinese Medicine Using Multi-class Support Vector Machines”.

## Authors’ contributions

FL, GL and XZ conceived the idea and designed the research. FL, YW and CZ acquired and processed the data. FL, ZX, CZ and GL performed the research and analyzed the results. FL, XZ, ZX and CZ wrote the paper. All authors read and approved the final manuscript.

## Pre-publication history

The pre-publication history for this paper can be accessed here:

http://www.biomedcentral.com/1472-6882/12/127/prepub

## Supplementary Material

Additional file 1 The clinical classification scale facial diagnosis of TCM. Click here for file

Additional file 2 Lip Image Feature Data. Click here for file

Additional file 3 84 Features Rank List and Items. Click here for file
