# Supplementary material for: Computer-assisted lip diagnosis on traditional Chinese medicine using multi-class support vector machines
Source: BMC Complement Altern Med. 2012 Aug 16;12:127. doi: 10.1186/1472-6882-12-127 (PMC3522569; doi:10.1186/1472-6882-12-127)
Supplement: Additional file 1 — The clinical classification scale facial diagnosis of TCM. [file 1472-6882-12-127-S1.doc]

Appendix A:

**The clinical classification scale facial** diagnosis of TCM

| code |  | | name | | | |  | | | | gender |  |
| --- | --- | --- | --- | --- | --- | --- | --- | --- | --- | --- | --- | --- |
| years |  | | height | | | |  | | | | weight |  |
| Classification of facial color | | looming reddish yellow , implicit bright and moist | | | | | | | | | | |
|  |  | cyan | ①cyan | | ②cyan black | | | ③cyan yellow | | |  | |
|  |  | red | ①pale red | | ②red | | | ③darkred | | | ④flushing malar | |
|  |  | yellow | ①pale yellow | | ②yellow | | | ③languishing and yellow | | | ④yellow and fat | ⑤yellow in forehead |
|  |  | white | ①pale white | | ②white | | | ③gleaming white | | | ④pallor | |
|  |  | black | ①black | | ②yellowish black | | |  | | |  | |
| Lip color | | ①red | ②deep-red | | ③Pale | | | ④Purple | | | | |
| gloss | | ①glossy | ②little glossy | | ③no glossy | | | ④dull orbit | | | | |
| texture | | freckles | | acne | | | | | other | | | |
| Part of face | | eye | cheek | | | forehead | | | | nose | | chin |
| General description | |  | | | | Doctor/date | | | |  | | |
