# Supplementary material for: Computer-assisted lip diagnosis on traditional Chinese medicine using multi-class support vector machines
Source: BMC Complement Altern Med. 2012 Aug 16;12:127. doi: 10.1186/1472-6882-12-127 (PMC3522569; doi:10.1186/1472-6882-12-127)
Supplement: Additional file 3 — 84 Features Rank List and Items. [file 1472-6882-12-127-S3.doc]

Additional file 1:

Feature Ranked List:

9 7 31 23 21 30 56 22 34 26 19 24 11 8 10 12 2 33 29 1 25 32 28 73 20 48

62 81 69 74 78 35 42 46 5 63 3 17 13 66 67 15 4 82 79 37 59 57 76 55 60

83 54 84 41 18 80 16 70 77 65 71 47 51 64 6 53 75 44 14 40 38 49 27 68

45 72 39 50 36 43 52 58 61

| No. | Color Features | No. | Haralick features | No. | Zernike moment features |
| --- | --- | --- | --- | --- | --- |
| 1 | Hue ( H ) | 25 | angular second moment | 38 | Zernike 1 |
| 2 | hue variance (HV) | 26 | contrast | 39 | Zernike 2 |
| 3 | Saturation ( S ) | 27 | correlation | 40 | Zernike 3 |
| 4 | saturation variance (SV) | 28 | sum of squares | 41 | Zernike 4 |
| 5 | Intensity ( I ) | 29 | inverse difference moment | 42 | Zernike 5 |
| 6 | intensity variance (IV) | 30 | sum average | 43 | Zernike 6 |
| 7 | Red ( R ) | 31 | sum variance | 44 | Zernike 7 |
| 8 | red variance (RV) | 32 | sum entropy | 45 | Zernike 8 |
| 9 | Green ( G ) | 33 | entropy | 46 | Zernike 9 |
| 10 | green variance (GV) | 34 | difference variance | 47 | Zernike 10 |
| 11 | Blue ( B ) | 35 | difference entropy | 48 | Zernike 11 |
| 12 | blue variance (BV) | 36 | information measures of correlation | 49 | Zernike 12 |
| 13 | Y (represents the intensity) | 37 | maximal correlation coefficient | 50 | Zernike 13 |
| 14 | Y variance (YV) |  |  | 51 | Zernike 14 |
| 15 | I (represents the color information) |  |  | 52 | Zernike 15 |
| 16 | I variance (IV) |  |  | 53 | Zernike 16 |
| 17 | Q (represents the color information) |  |  | 54 | Zernike 17 |
| 18 | Q variance (QV) |  |  | 55 | Zernike 18 |
| 19 | Y |  |  | 56 | Zernike 19 |
| 20 | Y variance (YV) |  |  | 57 | Zernike 20 |
| 21 | Cb |  |  | 58 | Zernike 21 |
| 22 | Cb variance (CbV) |  |  | 59 | Zernike 22 |
| 23 | Cr |  |  | : |  |
| 24 | Cr variance (CrV) |  |  | 84 | Zernike 47 |
